# Frequency and phenotype of type 1 diabetes in the first six decades of life: a cross-sectional, genetically stratified survival analysis from UK Biobank

**DOI:** 10.1016/S2213-8587(17)30362-5

**Published:** 2018-02

**Authors:** Nicholas J Thomas, Samuel E Jones, Michael N Weedon, Beverley M Shields, Richard A Oram, Andrew T Hattersley

**Affiliations:** a Institute of Biomedical and Clinical Science, University of Exeter Medical School, Exeter, UK

## Abstract

**Background:**

Type 1 diabetes is typically considered a disease of children and young adults. Genetic susceptibility to young-onset type 1 diabetes is well defined and does not predispose to type 2 diabetes. It is not known how frequently genetic susceptibility to type 1 diabetes leads to a diagnosis of diabetes after age 30 years. We aimed to investigate the frequency and phenotype of type 1 diabetes resulting from high genetic susceptibility in the first six decades of life.

**Methods:**

In this cross-sectional analysis, we used a type 1 diabetes genetic risk score based on 29 common variants to identify individuals of white European descent in UK Biobank in the half of the population with high or low genetic susceptibility to type 1 diabetes. We used Kaplan-Meier analysis to evaluate the number of cases of diabetes in both groups in the first six decades of life. We genetically defined type 1 diabetes as the additional cases of diabetes that occurred in the high genetic susceptibility group compared with the low genetic susceptibility group. All remaining cases were defined as type 2 diabetes. We assessed the clinical characteristics of the groups with genetically defined type 1 or type 2 diabetes.

**Findings:**

13 250 (3·5%) of 379 511 white European individuals in UK Biobank had developed diabetes in the first six decades of life. 1286 more cases of diabetes were in the half of the population with high genetic susceptibility to type 1 diabetes than in the half of the population with low genetic susceptibility. These genetically defined cases of type 1 diabetes were distributed across all ages of diagnosis; 537 (42%) were in individuals diagnosed when aged 31–60 years, representing 4% (537/12 233) of all diabetes cases diagnosed after age 30 years. The clinical characteristics of the group diagnosed with type 1 diabetes when aged 31–60 years were similar to the clinical characteristics of the group diagnosed with type 1 diabetes when aged 30 years or younger. For individuals diagnosed with diabetes when aged 31–60 years, the clinical characteristics of type 1 diabetes differed from those of type 2 diabetes: they had a lower BMI (27·4 kg/m^2^ [95% CI 26·7–28·0] *vs* 32·4 kg/m^2^ [32·2–32·5]; p<0·0001), were more likely to use insulin in the first year after diagnosis (89% [476/537] *vs* 6% [648/11 696]; p<0·0001), and were more likely to have diabetic ketoacidosis (11% [61/537] *vs* 0·3% [30/11 696]; p<0·0001).

**Interpretation:**

Genetic susceptibility to type 1 diabetes results in non-obesity-related, insulin-dependent diabetes, which presents throughout the first six decades of life. Our results highlight the difficulty of identifying type 1 diabetes after age 30 years because of the increasing background prevalence of type 2 diabetes. Failure to diagnose late-onset type 1 diabetes can have serious consequences because these patients rapidly develop insulin dependency.

**Funding:**

Wellcome Trust and Diabetes UK.

## Introduction

Type 1 diabetes is caused by autoimmune destruction of pancreatic β cells in genetically predisposed individuals and results in severe insulin deficiency with a requirement for treatment with insulin. It is typically considered a disease of childhood and adolescence, but can occur at any age. Type 2 diabetes is predominantly a disease of adulthood and is associated with obesity, insulin resistance, and relative but not absolute insulin deficiency. Type 2 diabetes is initially treated with lifestyle measures and oral glucose-lowering drugs.

Correct diagnosis of type 1 diabetes in young people (<20 years) is usually straightforward because it accounts for most (≥85%) cases of diabetes in that population.^1^ By contrast, identification of type 1 diabetes in adults older than 30 years is challenging because of the much higher prevalence of type 2 diabetes than type 1 diabetes in older adults (type 1 diabetes accounts for <5% of all cases).^2^, ^3^ Errors are often made when diagnosing type 1 diabetes later in life. For example, more than 50% of patients diagnosed with type 1 diabetes after age 35 years were shown to have type 2 diabetes in long-term follow-up.^4^, ^5^ Conversely, many older patients initially believed to have type 2 diabetes because of their age at diagnosis deteriorate rapidly and are subsequently found to have type 1 diabetes.^6^ Few studies^2^ have investigated how frequently type 1 diabetes presents in later life.

**Research in context**

**Evidence before the study**

Type 1 diabetes affects people of all ages, but data on the epidemiology of type 1 diabetes in adulthood are scarce. Most studies focus exclusively on type 1 diabetes in individuals who were diagnosed when aged 20 years or younger, which accounts for more than 85% of all cases and is easy to identify. Distinguishing between type 1 diabetes and type 2 diabetes in adulthood is more difficult because, in middle and old age, the incidence of type 2 diabetes increases sharply; thus, type 1 diabetes represents a small minority of cases in adulthood and recognition of these patients can be challenging. A systematic review by Diaz-Valenica and colleagues in 2015 emphasised the paucity of data on the incidence of type 1 diabetes in adults and encouraged the launch of epidemiological studies of adult-onset type 1 diabetes. The authors identified only ten studies reporting the incidence of type 1 diabetes after age 40 years. We repeated their systematic search of PubMed, Google Scholar, and Web of Knowledge for articles published up to April 20, 2017, and identified no additional studies. Considerable problems exist in the definitions of type 1 diabetes in older adults used in these studies: four studies solely relied on a clinical definition of insulin treatment by the clinician for classification as type 1 diabetes and only six studies used C-peptide or autoantibodies to confirm a diagnosis. However, use of autoantibodies has disadvantages: 1–7% of individuals without diabetes have positive autoantibodies depending on the cutoff used, and 1–7% of individuals with type 2 diabetes will have false-positive antibody results. Therefore, in older adults, in whom type 1 diabetes is rare (2–5% of all diabetes cases), patients with diabetes who are positive for autoantibodies are as likely to be false positive with type 2 diabetes as true positive with type 1 diabetes.

**Added value of this study**

We used a genetic, population-based approach to classify diabetes type over a broad age range and to compare two groups differing only by their genetic susceptibility to type 1 diabetes. We defined type 1 diabetes genetically as any excess of diabetes in the group with high type 1 diabetes genetic susceptibility compared with the group with low type 1 diabetes genetic susceptibility. Type 1 diabetes was shown to present throughout the first six decades of life, with 42% of type 1 diabetes occurring after age 30 years, representing 4% of all patients with diabetes diagnosed after age 30 years. We also examined the clinical characteristics of the patients with type 1 diabetes and found that the group diagnosed when aged 31–60 years had similar clinical characteristics and risk of diabetic ketoacidosis to the group diagnosed when aged 30 years or younger.

**Implications of all the available evidence**

Late-onset type 1 diabetes is difficult to diagnose in people aged 31–60 years because it represents only a small minority of patients diagnosed with diabetes; its misdiagnosis as type 2 diabetes results in inappropriate treatment. Patients with older-onset type 1 diabetes have been excluded from almost every major study of the biology of type 1 diabetes and, hence, little is known about its aetiopathology.

Biomarkers, such as C-peptide and islet-specific autoantibodies, can be used to distinguish type 1 diabetes from type 2 diabetes, but are not routinely measured or completely discriminatory. Progression to absolute insulin deficiency, defined by measurement of serum C-peptide concentration, can be used to identify type 1 diabetes, but is only useful 3–5 years after diagnosis. No studies to date have tested C-peptide in sufficiently large cohorts of patients.^2^, ^7^ Autoantibodies to the islet antigen glutamic acid decarboxylase (GAD) have been used for diagnosis, but are only present in 70% of patients with type 1 diabetes.^8^ The low incidence (5%) of type 1 diabetes compared with type 2 diabetes in people older than 30 years means that the proportion of people who have a true-positive autoantibody test (about 3·5% of all cases) is similar to the proportion with a false-positive test (around 2·5% of all cases because the definition of antibody positive is based on a 97·5th centile cutoff for a population without diabetes). Therefore, slightly less than half of autoantibody-positive patients older than 30 years will have type 2 diabetes and not type 1 diabetes.^9^ Clinical diagnosis based on a patient's clinical features, such as age of diagnosis and BMI, does not have sufficient discriminatory power because of the low prevalence of type 1 diabetes. These difficulties in accurate diagnosis mean that, although older-onset type 1 diabetes is recognised, accurate assessment of the proportion of type 1 diabetes that occurs at older ages and identification of its clinical features is challenging.

Polygenic risk for disease is rarely used to identify disease in populations because most individuals with high genetic risk do not go on to develop the disease.^10^ The concept of absent genetic susceptibility is used clinically to exclude conditions such as coeliac disease^11^, ^12^ and ankylosing spondylitis.^13^ Genetic predisposition to type 1 diabetes is polygenic, well defined by large genome-wide association studies,^14^ and does not predispose to type 2 diabetes.^15^ Strong genetic susceptibility to type 1 diabetes is predominantly *HLA* mediated, with other associated variants modifying immune recognition and function. This genetic susceptibility can be captured by the typing of 10–30 single nucleotide polymorphisms as a type 1 diabetes genetic risk score.^16^, ^17^ We have previously shown that, although a high type 1 diabetes genetic risk score can occur in people with type 2 diabetes or without diabetes,^16^ very few individuals (<5%) with type 1 diabetes have a type 1 diabetes genetic risk score below the 50th centile of a non-diabetes control or type 2 diabetes population.^18^ Thus, half of the population has such a low genetic susceptibility to type 1 diabetes that any diabetes they have is probably not type 1 diabetes but type 2 diabetes. The half of the population with high susceptibility to type 1 diabetes can have either type 1 or type 2 diabetes. The number of type 1 cases will correspond to the excess of diabetes cases compared with the number of cases of type 2 diabetes in the half of the population with low genetic susceptibility.

In this study, we aimed to define type 1 diabetes based solely on genetic susceptibility to determine the number of cases of type 1 diabetes, relative proportion of all cases of diabetes, and clinical features of type 1 diabetes throughout the first six decades of life.

## Methods

### Study design and participants

Between 2006 and 2010, UK Biobank recruited more than 500 000 individuals aged 37–73 years (99·5% aged 40–69 years) from across the UK. Individuals were registered with the UK National Health Service and lived within 25 miles of one of 22 assessment centres. Recruitment was unselected to provide a population-based cohort.^19^

From the initial UK Biobank dataset, for our genetic susceptibility analysis, we selected unrelated individuals of white European descent who had genetic data available. People of white European descent were those who self-identified as white European and were confirmed as ancestrally white by use of principal-components analyses of genome-wide genetic information.^20^

### Genetic susceptibility testing

We established the type 1 diabetes genetic risk score using published variants^16^ known to be associated with risk of type 1 diabetes (appendix). All variants were present in the imputed genotype data in UK Biobank. We used tag variants rs2187668 and rs7454108 to establish the *HLA-DR* haplotype and hence calculate the *HLA*-haplotype component of each individual's score.^16^, ^21^ The *HLA-DR* haplotype score was added to the score of the remaining variants, generated by summation of the number of predisposing alleles for each variant multiplied by the natural log of the odds ratio. All variants were well imputed (minimum imputation *r*^2^ 0·99). The variant rs4948088 was out of Hardy-Weinberg equilibrium (p<1 × 10^−40^) and was thus excluded from the score, leaving a total of 29 variants included in the score.

### Diabetes outcomes

Features of diabetes, including age of diagnosis, insulin use within 1 year of diagnosis, and insulin use at enrolment to the study, were self-reported via an interactive questionnaire completed at baseline enrolment into UK Biobank. Self-reported diabetes diagnosis has been shown to have a high accuracy compared with use of primary health records.^22^ Anthropometric measurements were collected at baseline when participants attended the assessment centre. We used the BMI recorded in UK Biobank, calculated as (weight [kg]/height [m]^2^). Gender was self-reported. Results were coded as missing if individuals had answered “don't know” or “prefer not to answer”. Records with inconsistencies in the data were removed (appendix).

Admissions for primary or secondary diabetic ketoacidosis were identified by use of diagnosis codes from hospital admissions data between April 1, 1997, and March 31, 2015. Diagnoses were coded according to the International Classification of Diseases version 10 (appendix).

### Statistical analysis

To define type 1 diabetes excess, we ranked individuals by type 1 diabetes genetic risk score and then split them by the median score into a high genetic risk score group and a low genetic risk score group. The 50% cutoff for type 1 diabetes genetic risk score was 0·231, which corresponded to the fourth centile for type 1 diabetes (individuals with diabetes who were diagnosed when younger than 17 years and who progressed straight to insulin) in the Wellcome Trust Case Control Consortium cohort.^16^ We evaluated the number of cases of diabetes in the two groups using Kaplan-Meier analysis, examining the number of years without diabetes before diagnosis by a doctor (as per participant self-report). Participants without diabetes were censored at their age of enrolment into UK Biobank.

**Figure 1:**
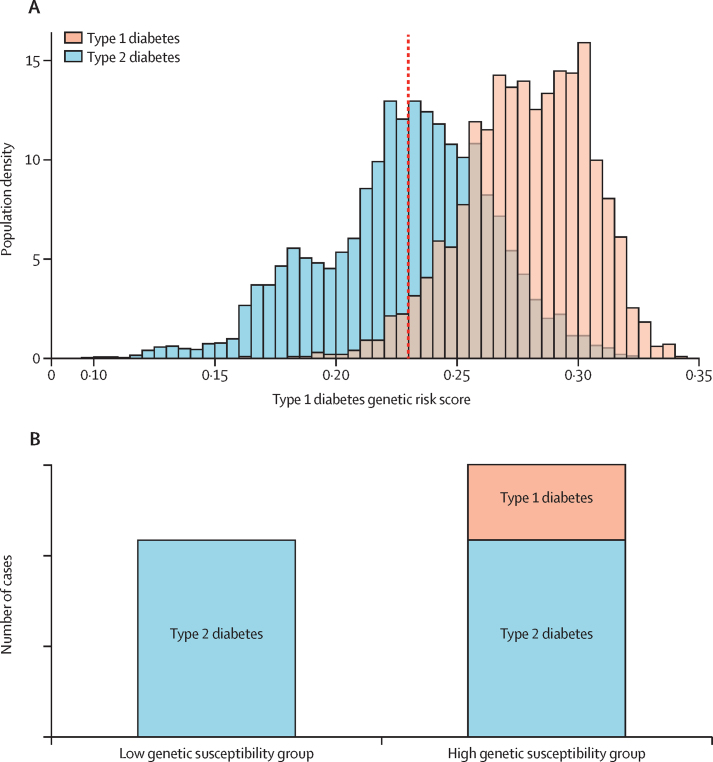
Use of a type 1 diabetes genetic risk score to establish the proportion of population with genetically defined type 1 diabetes (A) Distribution of type 1 genetic risk score in the Wellcome Trust Case Control Consortium type 1 diabetes and type 2 diabetes cohorts.^23^ Type 1 diabetes is restricted to high type 1 diabetes genetic risk scores. Numbers of individuals with type 2 diabetes above and below the 50th centile (dotted line; fifth centile for type 1 diabetes) will be the same; thus, any excess in the top 50% will be cases of type 1 diabetes. In a population where the proportions of type 1 diabetes and type 2 diabetes are unknown, this method can be used to determine the proportion of type 1 diabetes cases. (B) Schematic showing that the high type 1 diabetes genetic risk group will have the same amount of type 2 diabetes as the low type 1 diabetes genetic risk group in addition to an excess of diabetes contributed by type 1 diabetes.

Figure 1 shows how we used the type 1 diabetes genetic risk score to determine genetically defined type 1 diabetes. Almost all (96%) individuals with type 1 diabetes in the Wellcome Trust Case Control Consortium cohort^16^ have a genetic risk score above the 50th centile of the type 2 diabetes cohort, and, by definition, the numbers of type 2 diabetes cases above and below the 50th centile are equal. The type 1 genetic risk score in the general population has the same distribution and median as the type 2 diabetes population. Therefore, we genetically defined type 1 diabetes as the additional diabetes cases occurring in the 50% of the population with high genetic susceptibility to type 1 diabetes compared with the 50% of the population with low genetic susceptibility to type 1 diabetes. All remaining cases were defined as type 2 diabetes. For participants older than 60 years, data were censored at age 60 years because the overwhelming preponderance (>95%) of type 2 diabetes cases relative to type 1 diabetes cases in the diminishing at-risk population available for analysis rendered the subtraction method uninterpretable.^3^

Although we can determine the number of patients with type 1 diabetes from the excess, we could not use our method to establish individual diagnoses. However, we were able to derive summary characteristics for genetically defined type 1 diabetes. Categorical variables (eg, insulin treatment at 1 year) were recorded as the excess in the high type 1 diabetes susceptibility group compared with the low type 1 diabetes susceptibility group for each category. Continuous characteristics (eg, BMI) were derived by use of the mean value of the low susceptibility group (type 2 diabetes) and the mean of the high susceptibility group (combined type 1 and type 2 diabetes) to calculate a mean for the group with type 1 diabetes. For example, for BMI:

$${\bar{x}}_{T1D}^{BMI}=\frac{n_{H}{\bar{x}}_{H}^{BMI}-n_{L}{\bar{x}}_{L}^{BMI}}{n_{T1D}}$$

where *n*_H_, *n*_L_, and *n*_T1D_ represent the number of individuals and ${\bar{x}}_{H}^{BMI}$, ${\bar{x}}_{L}^{BMI}$, and ${\bar{x}}_{T1D}^{BMI}$ represent the mean BMI of the high, low, and excess groups, respectively. We derived summary statistics for the type 1 diabetes group from the means and SDs of the high and low genetic susceptibility groups for all normally distributed variables (apart from BMI) using standard algebra (appendix). Because of the bimodal distribution of BMI, we used the SD of the type 2 diabetes group when calculating the type 1 diabetes group's summary statistics.

We first compared the characteristics of type 1 versus type 2 diabetes in individuals diagnosed aged 31–60 years; we then compared the characteristics of type 1 diabetes in individuals diagnosed aged 30 years and younger versus those diagnosed aged 31–60 years. χ^2^ analysis was used for comparison of categorical characteristics. For continuous data, we used *t* tests to assess significance and calculated CIs for these summary statistics. Because seven clinical characteristics were tested, p values were adjusted for multiple testing using Bonferroni correction. All analyses were done with Stata, version 14.

### Role of the funding source

The funders of the study had no role in study design, data collection, data analysis, data interpretation, or the writing of the report. NJT had access to the raw data. The corresponding author had full access to all the data in the study and had final responsibility for the decision to submit for publication.

## Results

**Figure 2:**
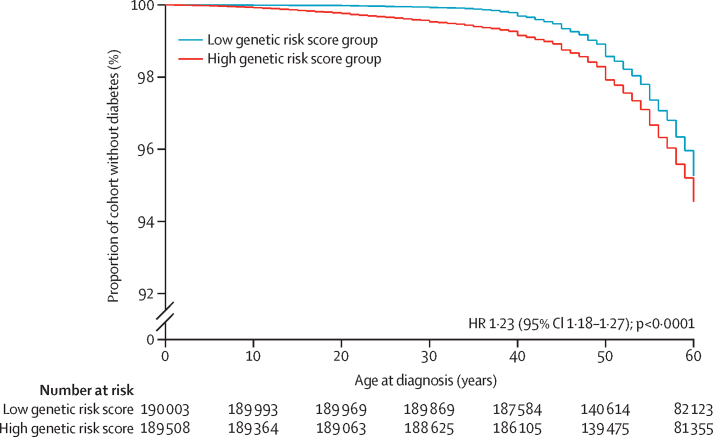
Kaplan-Meier curve of diabetes-free survival in the high and low genetic susceptibility groups Graph shows that diabetes was more likely in the high genetic susceptibility group. HR=hazard ratio.

We selected 379 511 unrelated individuals of white European descent from the initial UK Biobank dataset of 502 667 individuals with genetic data available. We ranked those individuals by type 1 diabetes genetic risk score and then split them around the median score into a high genetic risk score group (n=189 508) and a low genetic risk score group (n=190 003). 13 250 (3·5%) individuals had developed diabetes in the first six decades of life, 7268 of whom had a high type 1 diabetes genetic risk score and 5982 of whom had a low type 1 diabetes genetic risk score (figure 2). There was an excess of 1286 cases of genetically defined type 1 diabetes in the high genetic risk score group than in the low genetic risk score group (p<0·0001), which is consistent with 9·7% (95% CI 9·2–10·2) of all cases of diabetes being type 1 diabetes (figure 2).

**Figure 3:**
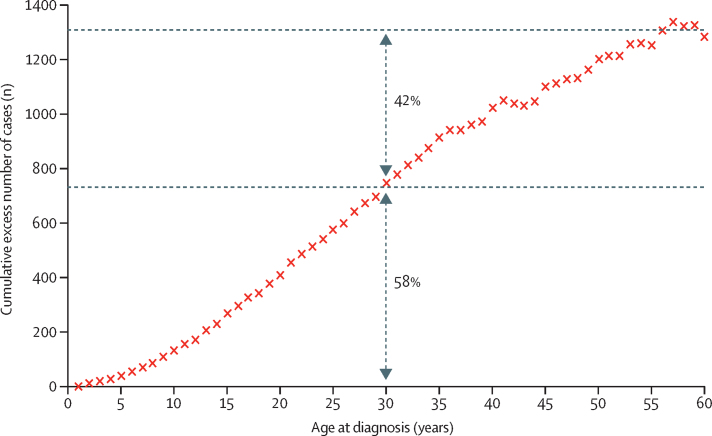
Cumulative excess of genetically defined cases of type 1 diabetes occurring throughout the first six decades of life 58% (749/1286) of type 1 diabetes cases were diagnosed when individuals were aged 30 years or younger; 42% (537/1286) were diagnosed when individuals were aged 31–60 years.

**Figure 4:**
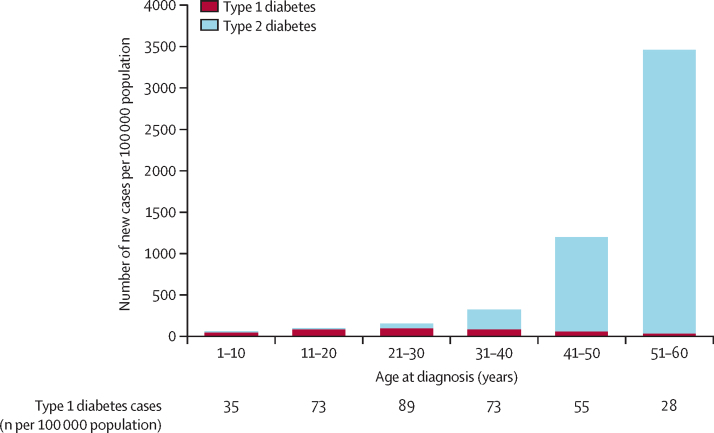
Incidence of genetically defined type 1 and type 2 diabetes in the first six decades of life Cases of type 1 diabetes were distributed across all ages of diagnosis, whereas cases of type 2 diabetes increased substantially with increasing age.

Cases of genetically defined type 1 diabetes were distributed across the first six decades of life (Figure 3, Figure 4). Of 1286 individuals with type 1 diabetes, 537 (42%, 95% CI 39–45) were diagnosed when aged 31–60 years and 749 (58%, 55–61) were diagnosed when aged 30 years or younger (p<0·0001). Type 1 diabetes accounted for 537 (4%, 4–5) of the total 12 233 diabetes cases diagnosed between ages 31 and 60 years and 749 (74%, 71–76) of the 1017 diabetes cases diagnosed aged 30 years or younger.

For diabetes diagnosed aged 31–60 years, clinical characteristics differed substantially between type 1 diabetes and type 2 diabetes (table 1). The group with type 1 diabetes had a lower BMI, was diagnosed at a younger age, and progressed to insulin more rapidly than the group with type 2 diabetes. For the group diagnosed aged 31–60 years, all 537 individuals (100%) with type 1 diabetes were being treated with insulin (at a mean of 14 years [95% CI 13–16] after diagnosis), whereas only 1924 (16%) individuals with type 2 diabetes were being treated with insulin (at a mean of 7 years [7–7] after diagnosis; p<0·0001). 61 (11%) of 537 individuals in the type 1 diabetes group had a recorded hospital admission for diabetic ketoacidosis compared with 30 (0·3%) of 11 696 individuals in the type 2 diabetes group (p<0·0001).

**Table 1:** Clinical characteristics of individuals with genetically defined type 1 and type 2 diabetes diagnosed aged 31–60 years

		**Type 1 diabetes (n=537)**	**Type 2 diabetes (n=11 696)**	**p value**
Age at study entry (years)		56 (55–57)	59 (59–59)	<0·0001
Age at diagnosis (years)		42 (41–43)	52 (52–52)	<0·0001
Sex				
	Male	335 (62%, 58–66)	7684 (66%, 64–67)	0·84
	Female	202 (38%, 34–42)	4012 (34%, 33–36)	0·84
BMI (kg/m^2^)		27·4 (26·7–28·0)	32·4 (32·2–32·5)	<0·0001
Insulin treatment				
	At 1 year after diagnosis	476 (89%, 86–91)	648 (6%, 5–6)	<0·0001
	At study entry	537 (100%, 100–100)	1924 (16%, 15–17)	<0·0001
Diabetic ketoacidosis as discharge diagnosis		61 (11%, 9–14)	30 (0·3%, 0·1–0·4)	<0·0001

Data are mean (95% CI) or n (%, 95% CI).

The clinical characteristics of type 1 diabetes in patients who had been diagnosed when aged 31–60 years were similar to the clinical characteristics of the disease in patients who had been diagnosed when aged 30 years or younger (table 2). Specifically, sex, BMI, insulin treatment at the time of enrolment into UK Biobank, and diabetic ketoacidosis as a discharge diagnosis were not different between the groups. A higher proportion of people in the 30 years or younger at diagnosis cohort received insulin within 1 year of diagnosis than in the cohort diagnosed at an older age.

**Table 2:** Clinical characteristics of patients with genetically defined type 1 diabetes, by age of diagnosis

		**Diagnosed aged 0–30 years (n=749)**	**Diagnosed aged 31–60 years (n=537)**	**p value**
Age at study entry (years)		53 (53–54)	56 (55–57)	<0·0001
Age at diagnosis (years)		19 (18–19)	42 (41–43)	<0·0001
Sex				
	Male	425 (57%, 53–60)	335 (62%, 58–66)	0·28
	Female	324 (43%, 40–47)	202 (38%, 34–42)	0·28
BMI (kg/m^2^)		26·7 (26·4–27·0)	27·4 (26·7–28·0)	0·14
Insulin treatment				
	At 1 year after diagnosis	723 (97%, 95–98)	476 (89%, 86–91)	<0·0001
	At study entry	741 (99%, 98–100)	537 (100%, 100–100)	0·14
Diabetic ketoacidosis as discharge diagnosis		65 (9%, 7–11)	61 (11%, 9–14)	0·70

Data are mean (95% CI) or n (%, 95% CI).

## Discussion

We used a novel genetically stratified survival analysis to identify cases of type 1 diabetes in a cross-sectional population of 379 511 people in UK Biobank. Genetically defined type 1 diabetes represented 9·7% (1286 of 13 250) of all diabetes cases diagnosed in the first six decades of life, with 58% (749 cases, 74% of all cases of diabetes in this age range) of type 1 diabetes cases diagnosed when patients were aged 30 years or younger and 42% (537 cases, 4% of all cases) of cases diagnosed when individuals were aged 31–60 years. The phenotype of type 1 diabetes was similar for the group diagnosed when aged 31–60 years and the group diagnosed when aged 30 years or younger; individuals in both groups had a low BMI, rapidly progressed to insulin treatment, and were at increased risk of diabetic ketoacidosis compared with participants with assumed type 2 diabetes. Our findings suggest that type 1 diabetes presents throughout the first six decades of life. Although type 1 diabetes is easy to identify and study in individuals aged 30 years or younger, identification of type 1 diabetes in individuals who present when older than 30 years is more challenging because of the much greater background incidence of type 2 diabetes in older populations.

We have shown that genetic predisposition to type 1 diabetes, defined in children, predisposes to diabetes at all ages. Our subtraction method allowed a genetic definition of disease. Although monogenic disease is usually defined by its genetic aetiology, to our knowledge, this study is the first to use a subtraction method with genetic predisposition to define cases of a common complex polygenic disease.

Numerous studies^2^ have shown that type 1 diabetes can occur in individuals older than 30 years, but researchers have been unable to quantify to what degree it occurs. Approaches to this dilemma have broadly categorised type 1 diabetes either by clinical definitions, C-peptide negativity, or autoantibody positivity. In clinical practice, further investigations are rarely done and, when they are, interpretation is challenging because of the high prevalence of type 2 diabetes in the population older than 30 years. Clinical diagnosis is often based on poorly discriminatory clinical characteristics and, as a result, misdiagnosis is common. Among individuals diagnosed with diabetes when older than 35 years, 56% of those who commence insulin treatment immediately do not progress to absolute insulin deficiency, and 7% of those not initially treated with insulin do progress to absolute insulin deficiency.^4^

The clinical characteristics resulting from a genetic definition of type 1 diabetes can be compared with the clinical characteristics of type 1 diabetes defined by the presence of islet autoantibodies. In children and young adults with diabetes, who are typically non-obese and rapidly insulin dependent, both genetically defined type 1 diabetes and islet-antibody-positive diabetes define almost all patients. We identified a similar phenotype when genetically defined type 1 diabetes was diagnosed in individuals aged 31–60 years: 89% of patients were treated with insulin within 1 year of diagnosis and about one in nine were admitted to hospital with documented diabetic ketoacidosis. By contrast, most patients with diabetes diagnosed in middle age who have islet antibodies do not usually proceed rapidly to insulin therapy. In latent autoimmune diabetes of adults (LADA)—defined as patients diagnosed when aged 35–70 years who have antibodies to GAD and who are not initially treated with insulin—about 30–40% of patients are treated with insulin within the first 3 years after diagnosis, and the BMI, lipid profiles, and genetic susceptibility of these patients are intermediate between young-onset type 1 diabetes and type 2 diabetes.^24^ Rather than a single intermediate phenotype, a possible explanation is that patients who are antibody positive when the prevalence of type 1 diabetes is low represent a mixture of true positives (young-onset type 1 diabetes phenotype) and false positives (type 2 diabetes). The phenotypic heterogeneity seen in LADA, depending on the population studied and the titre and number of antibodies used, supports this concept.^24^ Further research is needed to investigate genetically defined type 1 diabetes, autoantibodies, and C-peptide within a single cohort.

Our genetically stratified survival analysis approach to identifying type 1 diabetes in a population has several advantages. First, the permanence of genetic risk from birth allows survival analysis to be done in a cross-sectional study. Second, the survival curve for type 2 diabetes in the low genetic risk score group allows use of a sensitive, but not specific, cutoff to identify almost all type 1 diabetes cases and then remove type 2 diabetes cases by subtraction. Third, a genetic definition of type 1 diabetes at a population level identifies cases without making assumptions about associated clinical features or requiring autoantibody measurement. This approach meant that we were able to assess clinical features in our study because they were not used to define type 1 diabetes. This strategy of using type 1 genetic susceptibility is independent of and complementary to methods based on clinical features, autoantibodies, or C-peptide measurement. Notably, this method could potentially be applied to other polygenic diseases with strong genetic susceptibility to provide an unbiased population assessment of their contribution to a common phenotype with multiple alternative causes.

This study has clear clinical implications. Our findings alert clinicians that type 1 diabetes occurs often after age 30 years, but that it is difficult to detect because of the predominance of type 2 diabetes in older adults. A high index of suspicion for type 1 diabetes in later life is important because it has a rapidly progressive phenotype with a substantial risk of diabetic ketoacidosis. Diagnosis of type 1 diabetes should be considered in any middle-aged patient with type 2 diabetes who does not show good glycaemic control on rapidly escalating therapy, especially if they are slim. At the individual level, a high type 1 diabetes genetic risk score (above the median of the population) cannot be used to diagnose type 1 diabetes in a patient with diabetes diagnosed in middle age because most (>90%) individuals with a high score will have type 2 diabetes. By contrast, a low genetic risk score (below the median of the population) can be used to say that a patient is unlikely to have type 1 diabetes. This genetic exclusion method is also used in existing clinical guidelines for diagnosis of coeliac disease^11^, ^12^ and ankylosing spondylitis.^13^ Calculating the probability of a type 1 diabetes diagnosis for an individual will be helped in the future by development of statistical models that incorporate quantitative clinical features, such as age of diagnosis and BMI, with the type 1 diabetes genetic risk score.

This study has several limitations. UK Biobank is a voluntary study recruiting from 22 sites across the UK; these volunteers are typically healthier and of higher socioeconomic status than the general UK population.^25^ All participants have reached the age of 37 years, introducing a selection bias against people who are unhealthy or have not survived to this age. This selection bias might lead to an underestimate of type 1 diabetes at all ages and particularly in those diagnosed as children because they would be more likely to have serious morbidity or mortality from complications that could reduce likelihood of entry into the study. In our study, the prevalence of diabetes diagnosed in children younger than 10 years was 35 cases per 100 000 population, which is lower than expected from previous studies (around 100 cases per 100 000 population).^3^, ^26^ A further contributing factor is that UK Biobank participants were born between 1934 and 1971, and type 1 diabetes has been presenting at younger ages over time.^27^ Underestimation of type 1 diabetes diagnosed in the youngest decade would have reduced the proportion of cases diagnosed when aged 30 years or younger in our study. However, this underestimation will not alter the phenotypic similarity in the clinical characteristics of patients diagnosed in the two age groups or the finding that presentation after age 30 years is common.

The age distribution of people in UK Biobank meant that we only had power to assess diabetes in individuals aged 60 years or younger. Therefore, we cannot predict whether the frequency of type 1 diabetes diminishes after age 60 years. We limited the study to individuals of white European descent because the type 1 diabetes genetic risk score has only been validated in white people. This approach means the results are specific to this ethnic group, and other studies are needed to validate the type 1 diabetes genetic risk score in other ethnic groups and to investigate older-onset type 1 diabetes in these populations. We used a type 1 diabetes genetic risk score developed in a reference population who were all diagnosed with type 1 diabetes when younger than 17 years.^16^, ^23^ Although the type 1 diabetes genetic risk score has not been tested in an adult-onset type 1 diabetes population, genetic susceptibility to type 1 diabetes in later life almost completely overlaps with that of childhood diabetes.^28^ Large studies^16^, ^18^ of well defined type 1 and type 2 diabetes populations have shown that almost no overlap exists between risk of type 1 diabetes and risk of type 2 diabetes. Additionally, in the Wellcome Trust Case Control Consortium cohort, type 2 diabetes had an identical distribution of type 1 diabetes genetic risk scores to a control population without diabetes and a population with monogenic diabetes. These data support our assumption that the excess of cases in the high genetic risk score group are type 1 diabetes.

In conclusion, use of a novel genetic approach to define type 1 diabetes has shown that it presents across the first six decades of life and should not be considered a disease of children and young adults. Whatever age it presents, type 1 diabetes is associated with rapid requirement for insulin and risk of ketoacidosis, suggesting that it is not a milder phenotype if diagnosed later in life. A key area for both clinical practice and research in the future is to improve recognition of late-onset type 1 diabetes.
