# Supplementary material for: Frequency and phenotype of type 1 diabetes in the first six decades of life: a cross-sectional, genetically stratified survival analysis from UK Biobank
Source: Lancet Diabetes Endocrinol. 2018 Feb;6(2):122–9. doi: 10.1016/S2213-8587(17)30362-5 (PMC5805861; doi:10.1016/S2213-8587(17)30362-5)
Supplement: Supplementary appendix [file mmc1.pdf]

THE LANCET
Diabetes & Endocrinology

Supplementary appendix

This appendix formed part of the original submission and has been peer reviewed. We post it as supplied by the authors.

Supplement to: Thomas N J, Jones S E, Weedon M N, Shields B M, Oram R A, Hattersley A T. Frequency and phenotype of type 1 diabetes in the first six decades of life: a cross-sectional, genetically stratified survival analysis from UK Biobank. *Lancet Diabetes Endocrinol* 2017; published online Nov 30. http://dx.doi.org/10.1016/S2213-8587(17)30362-5.

Supplementary

Excluded cases

Subjects with diabetes who were likely to have filled inappropriately the age of diagnosis with the duration of diabetes were excluded. These 92 individuals who were stated to be diagnosed younger than 20 years of age (median age 10) and did not go straight to insulin treatment (78% were still not on insulin therapy). The alternative diagnosis would be childhood or adolescent Type 2 diabetes which was not recognised in children in the UK until 2000 and still is extremely rare in British White subjects [25].

Diabetes diagnosed by doctor?

There were four possible options: “yes”, “no”, “do not know” or “prefer not to answer”. 431 individuals who answered “do not know” or “prefer not to answer” were coded as missing.

Did you start insulin within one year of your diagnosis of diabetes?

Individuals were only shown this question if they answered “yes” to the previous question indicating that a doctor had told them they have diabetes. Female individuals who indicated they had diabetes only during pregnancy were not shown this question. The respondents who replied that they did have diabetes were then asked if they started insulin within a year, 93 Individuals who answered “do not know” or “prefer not to answer” were coded as missing.

What was your age when the diabetes was first diagnosed?

If the answer was more than the participants age it was rejected. If participants answered less than 10 they were asked to confirm.

Self-reported diabetes type

If the participant was uncertain of the type of illness they had, then they described it to the interviewer (a trained nurse) who attempted to place it within the coding tree. If the illness could not be located in the coding tree then the interviewer entered a free-text description of it. These free-text descriptions were subsequently examined by a doctor and, where possible, matched to entries in the coding tree. Two individuals were coded as missing who answered “yes” to having both T1D and T2D.

Hospital admission data

Data on hospital episode statistics (HES) for participants resident in England are provided to UK Biobank by the Administrative Data Liaison Service (ADLS). Records date back to 01 April 1997 and contain coded data on admissions, operations and procedures. UK Biobank has kept the data in its original format, according to HES specification. HES use the World Health Organization’s ICD (International Classification of Diseases and Related Health Problems) to record diagnosis information.

Calculating Clinical characteristics

Our group clinical characteristics were calculated as below.

$$\sigma_{AI}^2 = \frac{1}{N_{AI} - 1}\left([N_{ALL} - 1]\sigma_{ALL}^2 - N_{AI}\mu_{AI}^2 - [N_{NAI} - 1]\sigma_{NAI}^2 - N_{NAI}\mu_{NAI}^2 + [N_{ALL}]\mu_{ALL}^2\right)$$

σ_{AI}^2 is the variance of the autoimmune group

N_{AI} is the total number of patients in the autoimmune group

N_{NAI} is the total number of patients in the non-autoimmune group

N_{ALL} is the total number of patients in the whole

σ_{NAI}^{2} is the variance of the non-autoimmune group

σ_{ALL}^{2} is the variance of the whole group

μ_{AI} is the mean of the autoimmune group

μ_{NAI} is the mean of the non-autoimmune group

μ_{ALL} is the mean of the whole group

Supplementary Table 1: T1D SNPs included in the genetic risk score with weights. Effect allele is the risk increasing allele on the positive strand.

SNP	Gene	Odds Ratio	Weight	Effect Allele
rs2187668, rs7454108	DR3/DR4	48.18	3.87	
	DR3/DR3	21.12	3.05	
	DR4/DR4	21.98	3.09	
	DR4/X	7.03	1.95	
	DR3/X	4.53	1.51	
rs1264813	HLA_A_24	1.54	0.43	T
rs2395029	HLA_B_5701	2.5	0.92	T
rs3129889	HLA_DRB1_15	14.88	2.70	A
rs2476601	PTPN22	1.96	0.67	A
rs689	INS	1.75	0.56	T
rs12722495	IL2RA	1.58	0.46	T
rs2292239	ERBB3	1.35	0.30	T
rs10509540	C10orf59	1.33	0.29	T
rs4948088	COBL	1.3	0.26	C
rs7202877		1.28	0.25	G

rs12708716	CLEC16A	1.23	0.21	A
rs3087243	CTLA4	1.22	0.20	G
rs1893217	PTPN2	1.2	0.18	G
rs11594656	IL2RA	1.19	0.17	T
rs3024505	IL10	1.19	0.17	G
rs9388489	C6orf173	1.17	0.16	G
rs1465788		1.16	0.15	C
rs1990760	IFIH1	1.16	0.15	T
rs3825932	CTSH	1.16	0.15	C
rs425105		1.16	0.15	T
rs763361	CD226	1.16	0.15	T
rs4788084	IL27	1.16	0.15	C
rs17574546		1.14	0.13	C
rs11755527	BACH2	1.13	0.12	G
rs3788013	UBASH3A	1.13	0.12	A
rs2069762	IL2	1.12	0.11	A
rs2281808		1.11	0.10	C
rs5753037		1.1	0.10	T
